# Artificial intelligence in the management and treatment of burns: a systematic review

**DOI:** 10.1093/burnst/tkab022

**Published:** 2021-08-19

**Authors:** Francisco Serra E Moura, Kavit Amin, Chidi Ekwobi

**Affiliations:** Department of Plastic Surgery, Norfolk and Norwich University Hospital, Colney Lane, Norwich, NR4 7UY, UK; Department of Plastic Surgery, Manchester University NHS Foundation Trust, UK; Department of Plastic Surgery, Lancashire Teaching Hospitals NHS Foundation Trust, Royal Preston Hospital, Preston, UK; Department of Plastic Surgery, Manchester University NHS Foundation Trust, UK; Department of Plastic Surgery, Lancashire Teaching Hospitals NHS Foundation Trust, Royal Preston Hospital, Preston, UK; Department of Plastic Surgery, Lancashire Teaching Hospitals NHS Foundation Trust, Royal Preston Hospital, Preston, UK

**Keywords:** Artificial intelligence, Machine learning, Computer vision, Burn, Neural networks

## Abstract

**Background:**

Artificial intelligence (AI) is an innovative field with potential for improving burn care. This article provides an updated review on machine learning in burn care and discusses future challenges and the role of healthcare professionals in the successful implementation of AI technologies.

**Methods:**

A systematic search was carried out on MEDLINE, Embase and PubMed databases for English-language articles studying machine learning in burns. Articles were reviewed quantitatively and qualitatively for clinical applications, key features, algorithms, outcomes and validation methods.

**Results:**

A total of 46 observational studies were included for review. Assessment of burn depth (*n* = 26), support vector machines (*n* = 19) and 10-fold cross-validation (*n* = 11) were the most common application, algorithm and validation tool used, respectively.

**Conclusion:**

AI should be incorporated into clinical practice as an adjunct to the experienced burns provider once direct comparative analysis to current gold standards outlining its benefits and risks have been studied. Future considerations must include the development of a burn-specific common framework. Authors should use common validation tools to allow for effective comparisons. Level I/II evidence is required to produce robust proof about clinical and economic impacts.

**Highlights**

- We present a review of articles using machine learning in burns-related applications and discuss the future implications for the integration of AI into clinical practice.
- AI can assist clinicians in evaluating burn surface, diagnose burn depth, the need for surgery or other therapies, guide fluid resuscitation and predict complications and prognosis with a high degree of accuracy.
- A burn-specific framework reporting tool should be developed to ensure transparent, reproducible and ethical studies, including predictive accuracy in target setting.
- Randomised controlled trials and other forms of high-level evidence should be used to produce high-quality evidence about the clinical and economic impacts of using AI, ensuring its superior efficacy to traditional working routines.
- This new technology as envisioned will require extensive AI education and training of the clinician workforce and the public and the cultivation of a cross-disciplinary approach.

## Background

Burns are devastating injuries, representing a significant health burden to both the individual and the healthcare system. Victims of burn injuries present with considerable clinical, psychological and social sequelae. They are characterised as one of the most challenging presentations encountered in any trauma, necessitating input from specialists providing a variety of expertise. As such, they require specialist centers that can manage the provisions to facilitate holistic care. With burns being the fourth most common cause of trauma worldwide [1], artificial intelligence (AI) has vast potential to advance burn care by enhancing patient experience, improving population health, reducing cost and improving provider experience [2].

For an injury that is considered systemic, there is significant variability in its presentation and outcomes and stratifying data to improve outcomes poses a challenge. Still, unique to burns is that universally, large and frequently recorded datasets are accessible. As technology has advanced, there is an opportunity to bring about a paradigm shift towards improving outcomes for burn patients. IBM estimates approximately one million gigabytes of healthcare data are accumulated over a patient’s lifetime, with the data generated doubling every 2–5 years [3]. Electronic health records (EHRs) facilitate new ways to acquire and process meaningful information to inform clinical decision-making. Unfortunately, the raw analysis of these datasets is beyond the capabilities of traditional statistical methodology [4].

**Figure 1.**
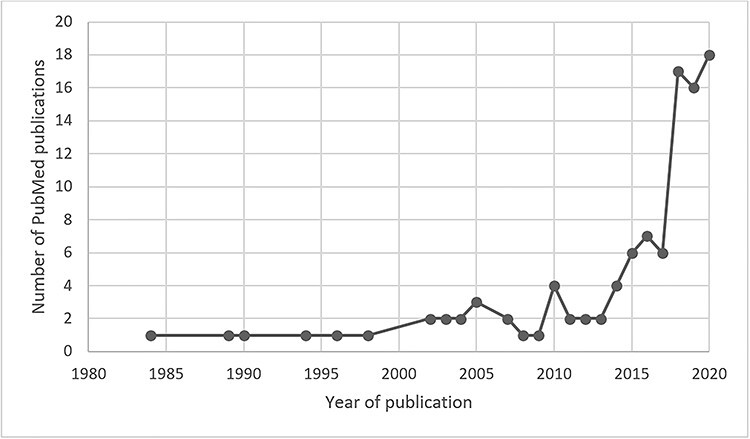
Trend of artificial intelligence burns-related publications on PubMed

AI holds promise for the care of patients with burn injuries and the interest in burn-related machine learning is growing exponentially (Figure 1). This review aims to: (1) provide a brief overview of the principal subfields of AI; (2) evaluate updated literature on AI applications that address the various aspects of burn care in the patient journey; (3) discuss the important considerations of machine learning and its implications in practice; and (4) highlight the role of the burn care professional in implementing successful AI technologies.

### Artificial intelligence

AI refers to intelligence demonstrated by machines in performing cognitive functions such as problem-solving, object/word recognition and decision-making [5]. Using statistical algorithms, models can accurately dissect meaningful outputs from large datasets [6]. The increasing power of computer processors makes it an opportune moment for medicine to embrace these novel technologies. In medicine, AI is starting to have a positive impact for clinicians (predominantly via rapid, accurate image interpretation), by improving workflow (to reduce medical errors), and for patients, by enabling their data to promote their own health [7].

**Figure 2.**
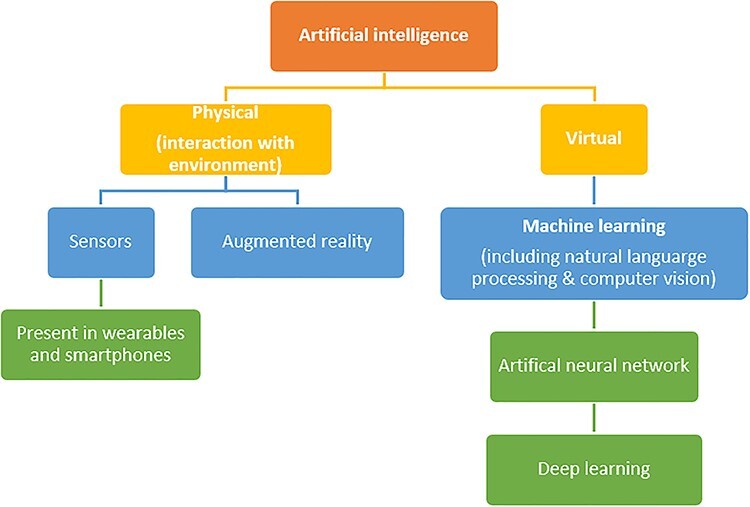
Domains of artificial intelligence. Physical artificial intelligence relates to machines interacting with their physical environment whereas virtual artificial intelligence is represented by machine learning

AI can be applied both physically or virtually [6] (Figure 2). The virtual domain refers to machine learning, neural networks, natural language processing and computer vision. Herein we summarise the virtual techniques pertinent to burns care.

#### Machine learning

**Figure 3.**
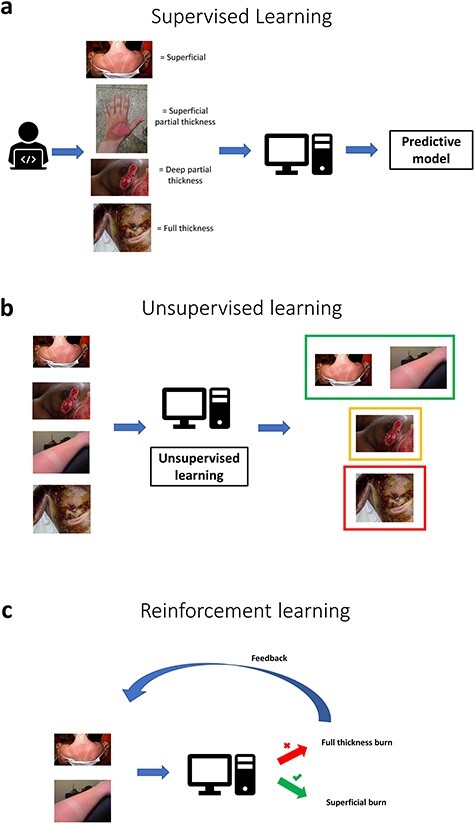
Illustration of different subtypes of machine learning. (**a**) Supervised learning involves the feeding of labelled data allowing the computer to create a predictive algorithm of a known output to correctly classify the depth of the burns. (**b**) Unsupervised learning uncovers any patterns such as the categorisation of burns depth from the unlabelled data. (**c**) Reinforcement learning is the process to successfully match the input and output data while learning from its successes and failures. It may share features of both a supervised and unsupervised process

Machine learning is a subset of AI that enables machines to make predictions by recognising patterns in structured data without explicit programming, but by using mathematical and statistical methods [8]. Machine learning, as opposed to conventional statistical analysis, is particularly useful for identifying subtle patterns in large datasets that may not be readily apparent to the human eye. Machine learning models have three principally distinct functions: supervised, unsupervised and reinforcement learning (Figure 3) [5]. Common mathematical algorithms used for supervised and unsupervised learning are defined in Table S1 (see online supplementary material).

**Figure 4.**
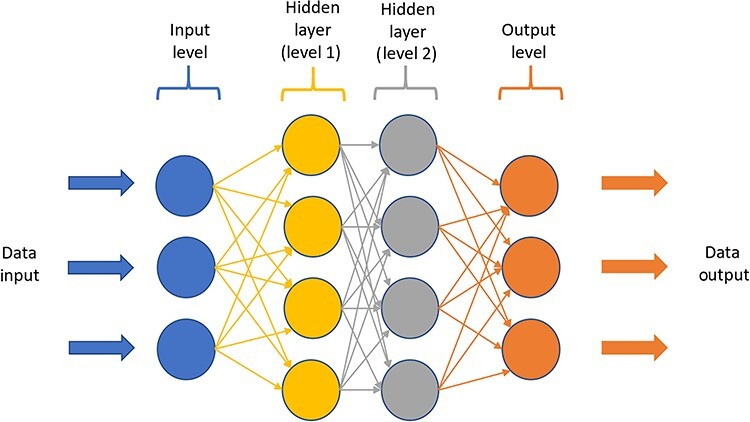
Representation of artificial neural networks. Artificial neural networks can independently process signals in layers of simple computational units. At the input level neurons receive information, perform a calculation and transmit output to the next neurone in the hidden layer. Within the hidden layers, calculations are carried out to analyse and extract the complex patterns in the dataset. The data is then passed onto the output layer that provides the final step in the analysis for interpretation. Deep learning involves the learning of more complex and subtle patterns than a simple one- or two-layer neural network

In supervised machine learning, an expert labels a training dataset which then trains an algorithm to predict a known output (Figure 3a) [8]. Supervised learning is often used for classification or regression problems, hence it can be used to predict from limited datasets of diagnoses or to estimate risk.

In contrast, unsupervised machine learning feeds unlabeled data to the algorithm and there are no known outputs to predict (Figure 3b) [8]. The objective is to find naturally occurring patterns with shared similarities within the unlabeled data.

Lastly, reinforcement machine learning is the process through which a program attempts to accomplish a task whilst learning from its successes and mistakes (Figure 3c) [8]. At present, reinforcement learning approaches are rarely employed in burn applications.

#### Neural networks

Advancing computational power has led to the creation of ‘deep learning’. Deep learning is a form of machine learning that studies data features using a multi-layered neural network. Neural networks are composed of computational units inspired by the neuronal connections that exist in animal brains. Artificial neural networks can be divided into three layers of neurons: input, hidden, output (Figure 4) [9]. The key differences between artificial neural networks and machine learning are that, firstly, the former requires significantly more data, and secondly, when fed raw data they learn their algorithms independently without the need for human intervention on which features to use [10].

Artificial neural networks cover several architectures, one of which is convolutional neural networks. A convolutional neural network, by definition, has one or more layers of convolution units. Therefore, convolutional neural networks reduce computational complexity and ensure translational invariance.

#### Computer vision

Computer vision uses mathematical techniques to analyse images or videos as quantifiable features (e.g. color, texture or position). Image segmentation is one of the major research areas in burns AI. The goal of image segmentation is to extract the region of interest in an image and disregard the background noise. This technique potentially allows burn depth evaluation, thus guiding decision making or estimating the time to healing. Computer vision has recently been integrated with machine learning techniques such as neural networks [11]. These methods have allowed computer vision to focus on higher-level concepts such as image-based analysis of patient cohorts, longitudinal studies and decision making within surgery [12]. In addition, there has been dramatic improvement, exceeding that of humans, in specific areas of image classification and recognition systems based on convolutional neural networks [13–16].

#### Natural language processing

Natural language processing focusses on the computer’s ability to analyse human language and speech. To successfully achieve a human-level understanding of language, natural language processing must expand beyond simple word recognition to extract meaning from texts into its analyses [17]. At present, natural language processing is rarely employed in burn-specific AI research. Still, this will undoubtedly play a fundamental role in burns. Chatbots can be especially useful in the follow-up and rehabilitation of burn patients to assess progress from a psychological, medical and administrative perspective.

#### Algorithm validation

The success of a machine learning algorithm depends on the features selected (e.g. urea concentration) and the performance criteria for training and validation [18]. It is critical that the algorithm created is reproducible and generalisable. Cross-validation processes allow the building of empirical data on many possible models, ultimately leading to the most generalisable algorithm. An assessment based solely on an initial validation test does not typically result in a generalisable model [19]. Hence, it is essential to include secondary or even tertiary external test sets (previously unseen by the model) to assess its true generalisability, thus providing patients with robust and safe models.

## Methods

**Figure 5.**
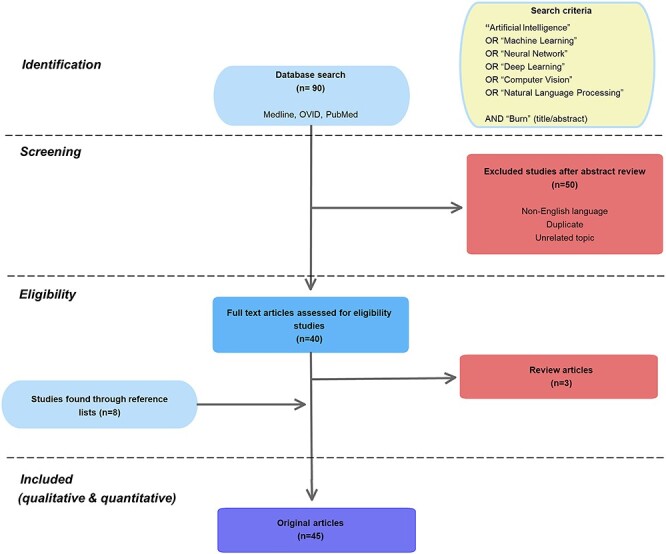
Flowchart showing systematic literature attrition

A comprehensive systematic search was carried out on Medline, Embase and PubMed to identify available literature related to burn care-related AI in September 2020. A combination of free text and medical subject headings terms were used (Figure 5). Search criteria included artificial intelligence, machine learning, neural network, deep learning, computer vision, natural language processing and burns.

Any English-language burn-related AI articles were eligible. Further relevant articles were identified from the references of published articles. Articles were excluded if the AI technology was not directly aimed at burn care. Articles in non-English language were excluded unless a valid English translation was available.

One author screened titles and abstracts for eligibility. All relevant full-text manuscripts were retrieved and studied. Eligible articles were classified according to their application in burn care.

Two authors independently collected data. Data collected included application to burns, population studied, algorithms and key features used, results (area under the receiver operating characteristic curve (AUC-ROC) and overall accuracy) and the validation method.

Descriptive statistics were used to summarise the data.

## Results

All original machine learning articles are summarised according to their burn application: survival/mortality (Table 1) [20–27]; assessment of burn depth (Table 2) [28–52]; estimation of body surface area (Table 3) [53,54]; antibiotic response/sepsis (Table 4) [55–57]; other miscellaneous applications (Table 5) [58–62].

Of 48 articles retrieved, three were review articles [4,18,63] whilst the remaining 45 were original research articles. Most articles provided mainly level 3 or 4 evidence in the form of retrospective cohort studies [64]. There were only two prospective cohort studies with level 2 evidence [35,61]. All eligible studies were therefore prone to attrition, confounding and selection biases inherent in these study designs.

**Figure 6.**
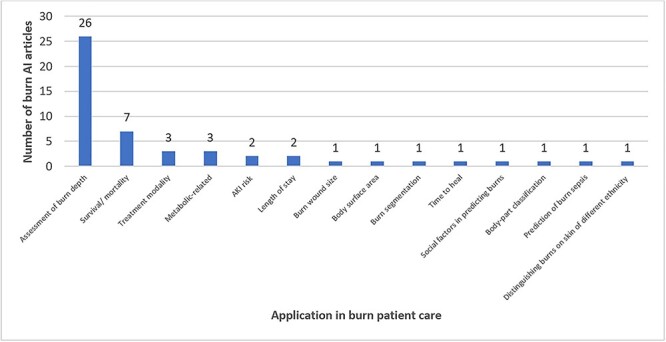
Number of machine learning articles on the different applications on burn patient care. *ML* machine learning

**Figure 7.**
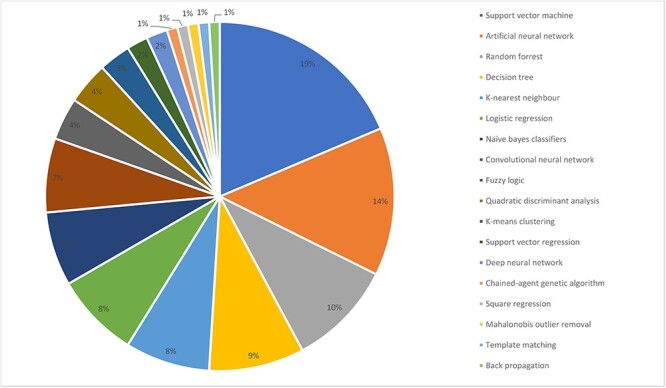
Algorithms used in burn-related machine learning articles

**Figure 8.**
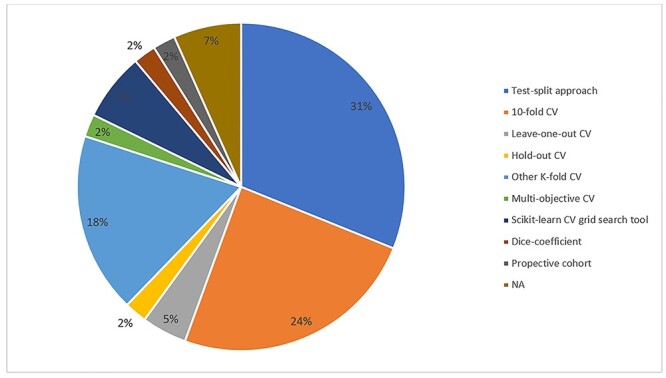
Methods of model validation used in burn-related machine learning articles.*CV* cross-validation

The most common applications of burn machine learning research were assessment of burn depth (*n* = 26) and survival/mortality (*n* = 7) (Figure 6). A diversity of algorithms were reported with the most frequent being support vector machines (SVMs) (*n* = 19) (Figure 7). Artificial and convolutional neural networks were increasingly described in recent literature in 14 and 7 articles, respectively. Machine learning algorithms are commonly tested using a *k-*fold cross-validation approach, with 10-fold being the most commonly applied (*n* = 11) as higher values of *k* lead to a less biased model (Figure 8) [18]. A significant proportion of articles did not report the use of any cross-validation tool. For instance, 14 articles (31%) solely used a test-split approach in which the complete data is divided into training and test data for the model. The *k-*fold approach differs from the test-split approach in that in the former data from the complete dataset can appear in both training and test datasets.

**Table 1:** Research articles using machine learning to predict survival/mortality in burn care

**Author (year)**	**Application**	**Population**	**Algorithm/tool**	**Key features**	**Results (AUC-ROC)**	**Results (accuracy)**	**Validation method**
Frye *et al*. [20] (1996)	Survival/mortality, LOS	1585 burn patients	ANN	Inhalation injury, age, TBSA	NS	Survival accuracy: 98% LOS accuracy: 72%	90% train; 10% test-split approach
Estahbanati and Bouduhi [21] (2002)	Survival/mortality	2096 burn patients	ANN	15 Different variables including inhalation injury, age, TBSA, etc	NS	Survival accuracy: 90% Sensitivity: 80%	75% training groups; 25% test-split approach
Patil *et al*. [22] (2011)	Survival/mortality	180 burn patients	NB, DT, SVM, back propagation	Age, gender, percentages of burns in eight areas of body	NB: 97.8% DT, SVM: 96.1% Back propagation: 94.9%	NB: 97.78% DT, SVM: 96.12% Back propagation: 95%	10-fold
Izamis *et al*. [23] (2012)	Metabolic indicators as marker of severity of burn	Burn on rat model *in vivo*	KMC, SVM, ANN, DT	Multiple biochemical markers	NS	VLDL and acetoacetate levels predict severity of burn with 88% accuracy	10-fold
Jimenez *et al*. [24] (2014)	Survival/mortality	99 burn patients	Fuzzy classifier, DT, NB, ANN	TBSA, infections, previous conditions	NS	CSR: 93%, Fuzzy classifier outperformed DT, NB, and ANN	Multi-objective
Stylianou *et al*. [25] (2015)	Survival/mortality	66,611 burn patients	LR, SVM, RF, NB, ANN	Age, TBSA, inhalation injury, comorbidities, type of burn	ANN had best mean with 0.971 Seldom significant statistical differences between LR and ML	NS	70% training; 30% test-split approach
Huang *et al*. [26] (2016)	Survival/mortality	6220 burn patients	LR, SVM	Gender, age, TBSA, inhalation injury, shock, period before admission	LR: 0.98 SVM correctly classified nearly 100% of cases	NS	1266 training; 549 testing; and 4405 validating samples
Cobb *et al*. [27] (2018)	Survival/mortality	31,350 patients	RF, SGB (DT)	Multiple patient and hospital characteristics	Patient-related factors: RF: 0.9 SGB: 0.92 Hospital-related factors: RF: 0.82 SGB: 0.62	NS	66% training; 34% test-split approach

*AUC-ROC* area under the receiver operating characteristic curve, *ANN* artificial neural network, *DT* decision tree, *KMC* k-means clustering, *LOS* length of stay, *LR* logistic regression, *ML* machine learning, *MSI* multi-spectral imaging, *NB* naïve bayes, *NS* not stated, *RF* random forest, *SVM* support vector machine, *SGB* stochastic gradient boosting, *TBSA* total body surface area.

**Table 2:** Research articles using machine learning to predict burn depth (classification)/treatment modality

**Author (year)**	**Application**	**Subjects**	**Algorithm**	**Key features**	**Results (AUC-ROC)**	**Results (accuracy)**	**Validation method**
Acha *et al*. [28] (2003)	Burn depth	62 burn images	FL, ANN	Digital color photographs (including lightness, hue, chrominance, etc)	NS	Average accuracy: 82%	5-fold
Acha *et al.* [29] (2005)	Burn depth	62 burn images	FL, ANN	Digital color photographs (including lightness, hue, chrominance, etc)	NS	Average classification rate: 82%	5-fold
Serrano *et al*. [40] (2005)	Burn depth	35 burn images	FL, ANN	Digital color photographs (including lightness, hue, chrominance, etc)	NS	Average classification rate: 88%	By group of experts from burns unit
Yeong *et al*. [66] (2005)	Burn healing time	41 burn images	ANN	Reflectance spectrometer	NS	<14 days accuracy: 96%, >14 days accuracy: 75%	10-fold
Wantanajittikul *et al.* [46] (2011)	Burn depth	5 burn images (34 sub-images)	NB, k-NN, SVM	Color and texture (contrast, homogeneity)	NS	SVM classification rate: 89.29% PPV: 0.92 Sensitivity: 0.84 Blind test classification: 75.33%	4-fold
Acha *et al*. [47] (2013)	Burn depth and treatment modality	62 burn images	k-NN	Digital color photographs (lightness, hue, chrominance)	NS	Burn depth classification (3 depths) rate: 66.2% Graft vs. non-graft classification rate: 83.8%	20 images training; 74 images test-split approach
Suvarna and Niranjan [48] (2013)	Burn depth	120 burn images	k-NN, SVM, TM	Digital color photographs (luminance, hue, chrominance)	NS	SVM outperformed other algorithms with an efficacy of 90%	3-fold validation
Ganapathy *et al*. [49] (2013)	Burn depth	7 burn images from *in vivo* porcine models	NB	Skin structure (thickness, perfusion) using OCT and PSI	0.87	NS	Immunohistochemical analysis
Li *et al*. [50] (2015)	Burn depth	18 Burn images from porcine skin model	SVM, k-NN	MSI (absorbance of different light wavelengths)	NS	Accuracy: 76%, matching the clinical judgment of expert burn surgeons	10-fold
Serrano *et al*. [51] (2015)	Burn depth and treatment modality	74 burn images	SVM	Digital color photographs (chroma, hue, kurtosis, variance of hue, etc.)	NS	Ability to heal accuracy: 80% Sensitivity: 0.97 Specificity: 0.60	20 images training; 74 images test-split approach
Badea *et al.* [52] (2016)	Burn depth	611 pairs (color and infrared) from 55 pediatric patients	CNN (ResNet), Histogram of topical features (RF, SVM)	Infrared and color digital camera (hue-saturation, texture, RGB-Yuv)	NS	Ensemble method precision: 65.12%	74,763 patches for training/validation; 125,731 patches for test-split approach
Heredia-Juesas *et al.* [30] (2016)	Burn depth	28 burn images of swine model	QDA	Digital photograph (RI, MSI and PPG)	NS	Accuracy: 0.77	K-fold cross-validation (k = 14)
Tran *et al.* [67] (2017)	Burn depth	396 burn images	One class SVM	Digital color photograph	NS	Precision: 77.78%	50% training; 50% test-split approach
Wang *et al*. [31] (2017)	Burn depth	1557 burn images on porcine model	CAGA, SVR	Near-infrared spectroscopy (chromophore and structural information)	NS	CAGA-SVR-RBF has a satisfactory prediction result and the model is very stable.	520 samples training; 520 samples validation; 517 samples testing sit approach Hold-out validation
Kuan *et al.* [32] (2017)	Burn depth	164 burn images	20 Classification algorithms (LR, NB, DT, RF, SVM etc.)	Digital color photograph Color and texture (lightness, hue, chroma)	NS	Best result with simple logistic with average accuracy of 73.2%	10-fold
Heredia-Juesas et al. [33] (2018)	Burn depth (viable *vs* non-viable)	34 burn images	QDA, KMC	Digital photograph (RI, MSI and PPG)	NS	Improves accuracy by 23.7% in detection of non-viable skin burn compared to QDA alone	K-fold cross-validation (k = 14)
Heredia-Juesas *et al.* [34] (2018)	Burn depth (viable *vs* non-viable)	34 burn images	QDA Mahalanobis outlier removal	Digital photograph (RI and MSI)	NS	Mahalanobis outlier removal improves accuracy by 13.6% of detection of non-viable skin	K-fold cross-validation
Martinez-Jimenez *et al*. [35] (2018)	Burn depth and treatment modality	34 training burn patients and 22 prospective burn patient cohorts	RF, KMC	Infrared thermography	Conservative *vs* graft: 92.3	85.35% Classification accuracy Treatment modality 0.9 kappa co-efficient	Prospective independent cohort
Heredia-Juesas *et al.* [36] (2018)	Burn depth	12 burn images on two adult porcine models	QDA	Digital photograph (RI, MSI and PPG)	NS	Accuracy 0.76	K-fold cross-validation (k = 12)
Rangaraju *et al.* [37] (2019)	Burn depth	56 *ex vivo* burn wounds on porcine model	LR, RF and linear SVM	OCT (morphological information) and RS (biochemical information)	0.94	Accuracy: 85%	10-fold
Wang *et al*. [38] (2019)	Burn depth	9 burn wounds on porcine model	RSER-K-NN, k-NNR, SVR, RF	Near-infrared spectroscopy (chromophore and structural information)	NS	Average relative error- 7%	N/a
Yadav *et al.* [39] (2019)	Burn depth	74 burn images	SVM	LAB color space (hog, hue, chroma, kurtosis, skewness)	NS	82% Accuracy Sensitivity: 87.8 Specificity: 83	20 images training; 74 image test-split approach
Cirillo *et al*. [41] (2019)	Burn depth	23 pediatric burn images	CNNs (VGG-16, GoogleNet, ResNet-50, ResNet-101)	Smartphone digital color photographs (as per CNNs)	NS	ResNet-101 outperformed other CNNs with: accuracy: 91% specificity: 94%	10-fold
Rowland *et al*. [42] (2019)	Burn depth	*In vivo* porcine model	SVM	SFDI	NS	Burn severity at 24 h accuracy: 92.5%	10-fold
Jiao *et al*. [43] (2019)	Burn segmentation of different burn depths	150 burn images	CNNs (R101FA, R101A, IV2RA)	Smartphone digital color photographs (as per CNNs)	NS	R101FA accuracy: 84.51%	Dice coefficient
Chauhan and Goyal [44] (2020)	Burn depth	141 burn images +63 unseen burn images	SVM, CNN (ResNet50, VGG16 and VGG19)	Image color and texture	NS	Burn severity accuracy: 91.53% for unseen burn images Body part classification accuracy: 93% ResNET50 outperformed generic method in burn severity by 10.6%	50% training; 30% testing & 20% validation split-approach
Wang *et al*. [45] (2020)	Burn depth	484 wound images	CNN (ResNet50 model)	Image color and texture	Macro average: 0.95	Accuracy: 80%	70% training; 15% validation and 15% validation test-split approach

*AUC-ROC* area under the receiver operating characteristic curve, *ANN* artificial neural network, *CAGA* chained-agent genetic algorithm, *CNN* convoluted neural network, *DT* decision tree, *FL* fuzzy logic, *KMC* k-means clustering, *k-NN* k-nearest neighbor, *LR* logistic regression, *ML* machine learning, *MSI* multi-spectral imaging, *NB* naïve Bayes, *NS* not stated, *PPG* photoplethysmography, *PSI* pulse speckle imaging, *QDA* quadratic discriminant analysis, *SVM* support vector machine, *RF* random forest, *RI* real image, *RSER* rotational feature subspace ensemble regression, *SFDI* spatial frequency-domain imaging, *SVR* support vector regression, *TM* template matching

## Discussion

### Applications of AI in burn care

**Figure 9.**
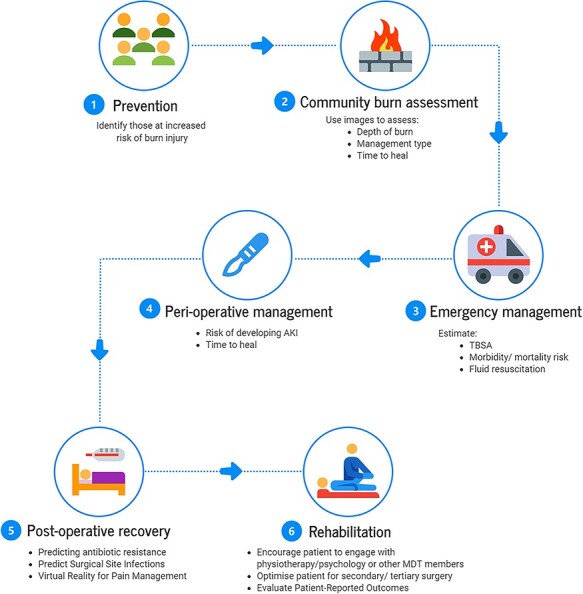
Possible benefits of artificial intelligence at the different stages of a patient with a burn injury. *AKI* acute kidney injury, *TBSA* total body surface area

AI has enormous potential to improve the experience of burn injury victims throughout their patient journey (Figure 9) besides improving the fundamental principles of burn care education.

As access to data increases, AI is becoming increasingly implicated in prevention, pre-/peri-/post-operative and rehabilitative care to guide procedural selection. It also has the potential to detect early complications. Herein we review some articles identified in the literature search pertinent to the different stages of the burn patient journey.

#### Prevention

Burn injuries are preventable. Advances in knowledge around the management and prevention of burns have led to a reduction in the mortality of burn injuries in high-income countries [65]. In contrast, this has not been applied satisfactorily in low- and middle-income countries. In higher-income countries, in which AI is likely to be employed first, this technology can allow patients to be risk-stratified by occupation, substance abuse, self-harm or other socio-economic factors that place patients at an increased risk of sustaining a burn injury. An algorithm able to identify this higher-risk group may prompt more regular community review or workplace inspection to mitigate any given risk.

Aghaei *et al*. studied the factors related to unintentional burns in children using data mining algorithms [60]. An artificial neural network-derived algorithm demonstrated better performance compared to SVM, random forest and logistic regression (Table S1). Most of the burn-related variables were related to individual social welfare status and their environments. Therefore, lessening the effects of these factors could reduce the incidence of pediatric burns. The same principles using AI can be applied to adult populations.

**Table 3:** Research articles using machine learning to predict body surface area/open wound size

**Author (year)**	**Application**	**Population**	**Algorithm/tool**	**Key features**	**Results (accuracy)**	**Validation method**
Liu *et al.* [53] (2018)	Predict open wound size	121 patients with >20% TBSA burns	DT, ANN, square regression	Multiple characteristics but key features included days since admission, fluid volume, TBSA burn, age	Combined ML models using four key features demonstrated >90% goodness of fit and < 4% absolute error	10-fold
Desbois *et al.* [54] (2020)	BSA	16 burn patients	DT, ANN	Height and circumferences of the bust, neck, hips, and waist	No significant difference between AI and the gold-standard (3D scans)	10-fold

*AI* artificial intelligence, *ANN* artificial neural network, *BSA* body surface area, *DT* decision tree, *ML* machine learning, *TBSA* total body surface area

#### Pre-hospital care

Given the limited and expensive nature of burn care resources, an automated system in local medical centers would be desirable to identify patients with burns requiring specialised input. AI technology can be useful to improve the standard of burn care for patients where burn experts may not be readily available. Despite several studies using AI as a tool to gauge burn depth, only three have specifically assessed treatment modalities [35,47,51]. Acha *et al*. [47] and Serrano *et al*. [51] explored the problem of burn color, image segmentation and classification using machine learning techniques. Acha *et al*. [47] used a multi-dimensional scaling approach to discover three discriminant features, including the degree of pink, texture and colorfulness of the burn image. This study demonstrated a 66% accuracy in classifying three varying depths of burns. Serrano *et al*. [51] proposed image pre-processing, and segmentation using color and texture features. A strict selection of texture features of burn wounds accurately classified 80% of burns healing ability. Martinez-Jimenez *et al*. combined infrared thermography with random forest and *k*-means clustering in a prospective study [35]. They report an accuracy of 85% in the treatment modality of the burn injury. These promising studies can be used to rationalise treatment and streamline early wound closure.

Machine learning has been used to determine healing time. Using reflectance spectrometry and artificial neural networks, Yeong *et al*. developed a model to predict whether a burn would take more or less than 14 days to heal using burn images, ultimately serving as a proxy for the assessment of burn depth for surgical planning [66]. The investigators reported an average predictive accuracy of 86%, suggesting that this method may serve as a superior alternative to direct visualisation. Although laser Doppler imaging (LDI) works differently by using a red diode laser to measure the extent of superficial dermal microvascular blood flow and thus estimate healing potential, combining LDI data with AI algorithms may potentiate the efficacy of this tool.

Digital photography is the most used method to gauge burn depth. To distinguish between burn wound/depth and healthy skin, Acha *et al*. used fuzzy logic and artificial neural network algorithms to extract hue-weighted saturation [29]. The use of digital photography for burn image segmentation and classification yielded an accuracy of 82%. Yadav *et al*. equaled this accuracy of depth using a SVM-based method, even though their model was not cross-validated, reducing its generalisability [39]. Tran *et al*. used a one-class SVM instead of the traditional SVM for burn image classification due to the imbalance degrees of burns data available [67]. The best classification results achieved using one-class SVM was 78% accuracy compared to 74% using traditional SVM. Since then, more powerful fine-tuned convolutional neural networks have been applied to burn depth. Tran *et al*. reported feasibility research on the role of convolutional neural networks in the categorisation of burn depth by extracting multi-color channels and converting these to binary values to improve algorithm performance [68]. Cirillo *et al*. have since described convolutional neural networks for image segmentation that can extract effective luminance-color texture for the classification of burn and non-burned areas [69]. Cirillo *et al*. have demonstrated an impressive 91% accuracy when analysing using deep convolutional neural network features obtained from pediatric burn injuries using high-performance digital cameras [41].

Multispectral imaging (MSI) techniques are remote sensing technologies that absorb different wavelengths. They have the ability to distinguish amongst varying severities of partial-thickness burns, which may consequently dictate the need for surgical intervention [70]. Li *et al*. combined a machine learning algorithm with MSI-acquired data, reporting a 76% accuracy in diagnosing burn depth, matching that of expert burn surgeons [50]. Similarly, another study described capturing burn images using non-invasive optical imaging (MSI, photoplethysmography, real image) to accurately classify 76% of burn depths using a quadratic discriminant analysis algorithm [36]. The same group used the same feature extraction technique and applied a tool which combined a supervised with an unsupervised classification algorithm, resulting in a 24% improvement in non-viable tissue detection [33].

Without uniquely relying on digital color differences, other reports analysed different factors in assessing burn depth. Ganapathy *et al*. assessed the skin thickness and perfusion information of *ex vivo* skin using optical coherence tomography and pulse speckle imaging [49]. Rangaraju *et al*. utilised optical coherence tomography and Raman spectroscopy to assess morphology and biochemical information, respectively [37]. The information on the collagen ratio classified burn depth with 85% accuracy. Badea *et al*. were the first to propose an ensemble method built upon the combination of standard classifiers and convolutional neural networks using thermal and color images for the purpose of burn severity assessment [52]. They reported an average precision of 65%. However, their approach required manual registration of images which would likely result in time delays. Rowland *et al*. accurately predicted burn severity (92.5% at 24 h) in animal burn models by combining SVM with spatial frequency-domain imaging [42].

Chauhan and Goyal [44] have reported the combination of standard digital photographs and machine learning to both categorise burn depth and the site of the body involved. The convolutional neural network algorithm outperformed the generic method in determining burn severity by 11%. Interestingly, Abubakar *et al*. [62] applied a fine-tuned convolutional neural network to distinguish burns from normal skin in both African and Caucasian images with a recognition accuracy of 97 and 99%, respectively. This research improves the generalisability of these advances to different populations. These results demonstrate the feasibility of utilising AI-assisted diagnosis in burn wounds.

**Table 4:** Research articles using machine learning to predict antibiotic response/sepsis in burn care

**Author (year)**	**Application**	**Population**	**Algorithm/tool**	**Key features**	**Results (AUC-ROC)**	**Results (accuracy)**	**Validation method**
Yamamura *et al.* [55] (2004)	Identifying severely ill patients and therapeutic aminoglycoside level	30 burn patients	ANN	Several physiological covariates, TBSA	NS	Multiple metrics, ANN outperformed LR	Leave-one-out
Yamamura *et al.* [56] (2008)	Aminoglycoside response against MRSA	25 burn patients	ANN, LR	Several physiological covariates, TBSA	NS	Multiple metrics, ANN outperformed LR	Leave-one-out
Tran *et al.* [57] (2020)	Burn sepsis	211 adult patients with burn TBSA >20%	Unsupervised learning (to identify best feature combinations) Supervised learning (LR, RF, SVM, NB, DNN, k-NN, XGBoost gradient boosting- DT)	Heart rate, body temperature, hemoglobin, BUN, and TCO_2_	k-NN: 0.96 LR: 0.945	k-NN: 89.7% LR: 0.868	Scikit-learn cross-validation grid search tool

*AUC-ROC* area under the receiver operating characteristic curve, *ANN* artificial neural Network, *BUN* blood urea nitrogen, *DNN* deep neural network, *DT* decision tree, *k-NN* k-nearest neighbor, *LR* logistic regression, *MRSA* methicillin-resistant *Staphylococcus aureus*, *NB* naïve Bayes, *NS* not stated, *RF* random forest, *SVM* support vector machine, *TBSA* total body surface area, *TCO_2_* total carbon dioxide

**Table 5:** Research articles using machine learning to predict other miscellaneous burn-related issues

**Author (year)**	**Application**	**Population**	**Algorithm/tool**	**Key features**	**Results (AUC-ROC)**	**Results (accuracy)**	**Validation method**
Yang *et al*. [58] (2010)	LOS	1080 burn patients	SVM, DT	Inhalation injury, age, gender, TBSA, various degrees of burns on body	NS	SVM outperformed DT and LR	10-fold
Tran *et al*. [59] (2019)	AKI risk	50 adult patients with >20% TBSA burn	K-NN	NGAL, urine output UOP, creatinine, NT-proBNP	NS	ML models using all key features achieved 90–100% accuracy	Scikit-learn cross-validation grid search tool
Aghaei *et al*. [60] (2019)	Factors related to unintentional burns	198 children with unintentional burns (case––control retrospective study)	SVM, ANN, RF, LR	BMI, socio-economic factors, etc.	ANN-MLP: 0.762 SVM-L: 0.731 RF: 0.751 LR-FS: 0.711	ANN-MLP: 0.733 SVM-L: 0.672 RF: 0.672 LR-FS: 0.672	70% training; 30% test-split approach
Rashidi *et al*. [61] (2020)	AKI risk	50 burn patients (retrospective) + 51 burn and non-burn patients (prospective)	LR, K-NN, RF, SVM, CNN	Age, gender, TBSA, NGAL, creatinine, NT-proBNP, and UOP	Models (DNN or LR) using NGAL and NT-proBNP: 92	Models (DNN or LR) using NGAL and NT-proBNP: 92 and 91%	Scikit-learn cross-validation grid search tool
Abubakar *et al*. [62] (2020)	Distinguishing burn from normal skin in different ethnicities	680 (Caucasian) and 270 (African) burn images	CNN (ResNet50)	Digital color photographs	NS	Recognition accuracy in: African dataset: 97.1% Caucasian dataset: 99.3%	80% Training; 20% validation test-split approach

*AKI* acute kidney injury, *AUC-ROC* area under the receiver operating characteristic curve, *ANN* artificial neural network, *BMI* body mass index, *CNN* convoluted neural network, *DNN* deep neural network, *DT* decision tree, *k-NN* k-nearest neighbor, *LOS* length of stay, *LR* logistic regression, *NGAL* neutrophil gelatinase associated lipocalin, *NT-proBNP* N-Terminal pro-B-type-natriuretic peptide, *NS* not stated, *RF* random forest, *SVM* support vector machine, *UOP* urine output, *TBSA* total body surface area

#### Peri-operative

AI technology can benefit patients requiring definitive reconstructive surgery. Pre-operatively a patient can be optimised for surgery by tracking weight, glucose and exercise via mobile applications [71–73]. Automated analysis of all pre-operative mobile and clinical data could provide a more patient-specific risk score for operative planning and yield valuable predictors for post-operative care.

Reliable diagnosis is supported by accurate estimation of the total body surface area (TBSA) and wound depth assessment, critical to treatment success. The precise quantification of TBSA by traditional measures such as Lund and Browder charts and ‘rule of palms’ have interobserver variability and are challenging when faced with asymmetrical injuries [66]. Machine learning can address this issue. An algorithm could rapidly and accurately predict TBSA by coupling burn images with their relevant TBSA. Consequently, more accurate resuscitation protocols could be generated in addition to surgical planning strategies for skin grafting. Jiao *et al*. [43] employed deep convolutional neural networks to automatically segment burn wounds using smartphone digital photographs. The results showed 84.51% accuracy in identifying varying depths across different TBSAs. Desbois *et al*. highlighted the importance of assessing the accuracy of morphology modelling since it can greatly affect TBSA [74]. The same research group then applied supervised machine learning algorithms in identifying key anthropometric measurements to build a 3D model showing good accuracy in estimating TBSA when compared to gold standard 3D scans [54]. On this note, it would be interesting to see how these results compare to that of clinicians when making clinical assessments.

Equally important are prognostic tools. Estahbanati and Bouduhi were one of the first to utilise artificial neural networks in predicting survival in burn patients with an accuracy reaching 90% [21]. They demonstrated that non-linear techniques are better suited to address complex questions regarding prognosis due to their ability to observe real events rather than evaluating the relative influence of variables on each other. Impressively, Huang *et al*. have published a successful robust model that correctly classified with an AUC-ROC of 99.5% of survival prognosis according to the admission characteristics [26]. In a slightly different application, Cobb *et al*. used random forest and decision trees to demonstrate that both patient and hospital factors are predictive of survival in burn patients [27]. This could help inform decisions about where burn patients should be treated. In contrast, only one article reported no AI advantage compared to traditional statistical methods, concluding that although some machine learning methods performed marginally better than LR, the differences were seldom statistically significant in predicting burn survival [25].

AI can be useful in pandemics (e.g. COVID-19). Data can be rapidly collated, and an algorithm derived to identify how such diseases impact survival and, as a result, how best to re-allocate resources. However, there is a paucity of literature on this topic at the current time.

Acute kidney injury (AKI) after burns has a significant impact on short- and long-term outcomes; thus, it is crucial to develop methods to identify patients at risk. Tran *et al*. developed used a K-nearest neighbor algorithm to predict AKI risk with 90–100% accuracy [75]. The same group [61] later compared machine learning models for early recognition of AKI using different laboratory findings in the predictive model. Their models accurately predicted AKI 62 h in advance.

Given the growing problems with antibiotic resistance, Tran *et al*. [57] have most recently published an algorithm with a 90% accuracy and an ROC-AUC of 0.96 in predicting sepsis secondary to large burns using key indicators such as blood urea nitrogen and hemoglobin. Additionally, Yamamura *et al*. [56] demonstrated the use of artificial neural networks in predicting the response to aminoglycoside antibiotics against methicillin-resistant *Staphylococcus aureus* in burn patients.

#### Post-operative

AI may prove beneficial to the patient in the post-operative period facilitating them in being at the center of their care. This technology can help patients monitor their progress regarding physiotherapy and set reminders to promote engagement and compliance. Likewise, machine learning can be useful to the healthcare team. For instance, predicting surgical site infections in the immunosuppressed burn patient. Machine learning has outperformed conventional statistics in the prediction of surgical site infections by building non-linear models that incorporate multiple data sources, including diagnoses, treatments and laboratory values [76]. Consequently, this can influence subsequent treatments including antibiotic therapy and dressing changes. Indeed, virtual reality (VR) has been shown to have a positive impact during change of dressings. Preliminary results demonstrated a reduction in pain after combat-related burn injuries during debridement using adjunctive immersive VR [77]. There remains considerable room for research within this area.

#### Rehabilitation

Rehabilitation after burn injuries can last for many years and it is essential that a multidisciplinary approach is sought. Rehabilitative programs play an essential role in decreasing post-traumatic stress and optimising functional independence. Patient-reported outcomes provide important patient-centred data to capture subjective datasets (impact of disease, treatment, quality of life measures). Digital platforms that monitor patient-reported outcomes could generate unique algorithms to screen symptoms, which would then not only alert the healthcare team but also automatically trigger a customised patient care plan. Merging AI technology with patient-reported outcomes offers the potential to improve outcomes and quality of care [78] as well as evaluating the efficacy of treatments [79].

Natural language processing has been used to examine EHRs to identify words and phrases in medical records that predicted anastomotic leak after colorectal resections [80]. These findings can be translated to the care of burns patients. Besides, the ability of algorithms to self-correct can increase the utility of their predictions as datasets grow to become more representative of a patient population.

#### Medical education

The evolution of technology has meant that reliance on memorising and retaining large volumes of information could become more obsolete with effort steered towards other skills. The system is likely to adapt, such that competence is likely to have communication skills, emotional intelligence and IT skills as part of its remit.

The use of VR in addition to real data-driven simulation will be fundamental to train the next generation of burns professionals. The acute nature of burn injuries generates a stressful environment. A skilful, communicative and well-coordinated team is key to managing such instances. Many adverse events in burns emergency are a consequence of non-technical skills, such as communication, leadership and teamwork. Thus, enhanced data from significant adverse events has the potential to make an impact on the acquisition of non-technical skills [81].

An example of innovation using AI to improve outcomes in settings which lack specialised skillsets or in less resourceful regions is *Proximie*. This is a secure, cloud-based augmented reality platform enabling real-time collaboration between a local (operating) surgeon and a remote (assisting) surgeon [82]. Both local and remote teams can communicate employing a two-way audio stream, using several integrated augmented reality features to further clarify the advice provided.

At present, there is no formal AI teaching curriculum. Healthcare students and trainees should be offered the opportunity to learn AI-related skills during electives, as part of leadership or business tracks, or in post-graduate research schemes. A new infrastructure for learning must be introduced, and educators from disciplines such as computer science, mathematics, ethnography and economics will likely play a key role. Another important aspect overlooked in medical training is EHRs, especially since they will assist in the implementation of AI in healthcare. It is imperative that less-developed countries share the same benefit from AI education.

### Current limitations of AI

An important concern regarding AI algorithms is their interpretability [83]. Whilst artificial neural networks address problems that may be missed by humans, its black box structure gives us little knowledge on how or why such an algorithm was developed. The accountability if a patient experiences harm from an AI-driven clinical decision remains unclear [84]. Liability may extend to the treating physician, hospital or software creator.

Reliable uptake in data collection has been and is a challenge for surgical teams. The quality of the algorithm depends upon the quality of data uploaded. Inherent systematic biases that occur in data collection, such as missing data, underrepresentation of women and racial minorities, may lead to inaccurate AI predictions [85]. Furthermore, current workflows are still operator dependent. The limited availability of high-quality data for training, correctly labelled with the outcome of interest, is a recurrent issue in this field. For instance, available data may be overrepresented with ‘interesting cases’ and not necessarily representative of the normal burn population, leading to a sample selection bias. This highlights the need for professionals to establish how and what common performance metrics need to be amassed, preferably at international level. With regards to missing data in the pre-processing imputation phase, extensive research into the use of statistical methods and machine learning imputation has been made to safely identify how to estimate these missing values in data processing and, where appropriate, disregard these values [86–88].

Burns is a discipline with great potential to exploit AI given the numerous multimedia comprising two- or three-dimensional imaging. Standardisation of these images, which includes angles and lighting, is not only difficult to achieve between different centers but is often inconsistent.

Data sharing and confidentiality is an ethical and bureaucratic challenge. Retrospectively gaining authorisation and making data anonymous will be difficult and open-source datasets are uncommon. Moreover, analyses of datasets that are fragmented will eventually lead to bias as described above. Yet the burns community, in particular, has continuously demonstrated their openness and willingness to embrace accumulative data through the development of the UK National Burn Injury Database and the International Burn Injury Database (IBID) [89].

Despite advances in the recognition of subtle patterns, AI is still limited by its inability to determine causal relationships and to deduce a clinical interpretation of its data analyses [90]. AI remains poor in appreciating the clinical context in which the relevant algorithm should be applied. Thus, clinicians must, as usual, critically evaluate AI predictions. AI will require further validation in the form of prospective observational studies and randomised controlled trials (RCTs) [18].

Even though the public has great enthusiasm for the concept of AI, some clinicians still have some resistance towards it. All explanations outlined above are valid points for such resistance, although there are others. The lack of basic and continuous education of clinicians regarding AI prevents a mass focus on radically developing this phenomenon. There is also the natural concern that AI will replace the role of physicians [91], although the mainstream opinion in the literature suggests that AI will complement physician intelligence [7]. Lastly, the lack of a legal framework, particularly in healthcare-related AI, leaves the physician exposed to potential legal repercussions when outcomes are not favorable [92]. Hence the need for proper validation.

The economic impact of AI must also be addressed. A systematic review proved that there are methodological deficits in the cost-effectiveness of AI in medicine and that future economic evaluations require a more comprehensive assessment to enable economic decisions for or against implementing AI in health care [93].

### Future considerations for the burn care provider

Clinicians will benefit from education in digital medicine to deal with the growing prospect and introduction of AI. The principles of AI engineering and digital health literacy should be implemented in graduate healthcare-related disciplines. Main burn organisations should encourage their members to research this topic by providing financial backing in the form of grants and by creating conferences with AI as the main theme. Equally, journals should encourage the submission of articles related to AI.

Despite the existing clinical data registries such as IBID, international and national burns organisations should adapt their existing databases to record relevant data that will be useful for patient management. Creating a database that carries the entirety of burns’ experience, which is representative of the population with appropriate representation of gender, age, ethnic and socio-economic background may lead to a technology that allows real-time clinical decision support. As stakeholders in the adoption of AI-technologies, burn surgeons should partner with data scientists and statisticians to ensure the right questions are answered. On this note, establishing who has access to these datasets is key. Each nation should address the issue of data protection and ownership ensuring that confidentiality is respected, not only medicolegally but also to ensure that confidence is maintained from the general public in the medical profession. There is a consensus to move towards patient ownership of data as this is likely to result in increased patient engagement [94].

To allow for the full benefit of AI, clinical validation tools should be developed to authenticate firstly the core concepts of AI and secondly the clinically relevant outcomes so these can be safely applied in practice. Important steps have been taken towards this with a report that proposes a framework to develop transparent, replicable, ethical and effective research in healthcare AI [95]. Clear and common validation guidelines should be followed by authors to ensure that their algorithms have been appropriately tested and are thus comparable before being accepted for publishing by journals. Also, it is important to consider the specificity and sensitivity of these models, which will have a clinical impact [96]. This has an important impact on prognostic modelling and decision-making.

## Conclusion

AI is an innovative and fast-moving field with remarkable potential in burn care to improve (1) the accuracy of diagnoses and treatment, (2) the efficiency of care, and (3) the workflow of healthcare professionals. The burn patient must be managed in a holistic manner when assessing and employing innovative technologies but there is an onus to ensure clinical implementation should only be carried out with robust clinical validation. A burn-specific framework reporting tool should be developed to ensure transparent, reproducible and ethical studies, including predictive accuracy in target setting. Subsequent prospective feasibility tests and RCTs should be used to produce high-quality evidence about their clinical and economic impacts, ensuring its superior efficacy to traditional working practices. Once adequate high levels of evidence have been attained, survival and morbidity AI appears to be the most advanced application in burn care at present, which could potentially be used as an adjunct to clinical practice. Further investigation should be carried out in the ability to predict burn depth and the ability to heal using imagery, as this domain presents the most promise to the medical community, in particular with regard to resource allocation. Patients and clinicians who are willing to take charge using digital means and algorithms should be empowered. At present, there are exaggerated claims about the superiority of AI over clinicians, which poses a risk for patient safety, population health and confidence in the medical profession. Data sharing and ownership including that of multimedia using clinical data registries should be made clear. This new technology as envisioned will require extensive AI education and training of the clinician workforce and the public and the cultivation of a cross-disciplinary approach that includes data scientists, computer scientists and engineers, in addition to pharmacists, nurses, physiotherapists, psychologists and doctors, to generate meaningful interpretation of data. With the large volume of burn data, AI can assist clinicians in evaluating burn surface, diagnose burn depth, the need for surgery or other therapies, guide fluid resuscitation, and predict complications and prognosis with a high degree of accuracy. Education and encouragement of AI technologies are key to delivering burns care on a far more rational, efficient and tailored basis. However, it cannot replace the art of caring.

## Supplementary Material

Supplement_Table_1_tkab022

## Data Availability

All data is presented in the article.

## Abbreviations

AI: Artificial intelligence; AKI: Acute kidney injury; AUC-ROC: Area under the receiver operating characteristic curve; EHRs: Electronic health records; IBID: International burns injury database; MSI: Multispectral imaging; SVM: Support vector machines; TBSA: Total body surface area; VR: Virtual reality
