# Supplementary material for: Artificial intelligence in the management and treatment of burns: a systematic review
Source: Burns Trauma. 2021 Aug 19;9:tkab022. doi: 10.1093/burnst/tkab022 (PMC8375569; doi:10.1093/burnst/tkab022)
Supplement: Supplement_Table_1_tkab022 [file supplement_table_1_tkab022.docx]

# **Supplement Table 1.** Common Machine Learning Algorithms used in Burn-related Artificial Intelligence.

| **Machine Learning Problem** | **Algorithm** | **Explanation** | **References** |
| --- | --- | --- | --- |
| Supervised Learning | Decision Tree | A flowchart structure that helps make binary decisions about non-parametric data items that can be used both for classification and regression problems | [97] |
|  | Fuzzy Logic | FL is the science of reasoning, thinking, and inference. This is a data-handling tool that instead of assuming everything is black and white, it allows ambiguity by recognising that most things fall somewhere in between | [98] |
|  | K-Nearest Neighbour | A popular non-parametric clustering algorithm used for data classification and regression that is based on the number of k-neighbours | [99] |
|  | Logistic Regression | A parametric technique that quantifies the relationship between a dependent categorical outcome and one or more independent predictor variables giving predicted probabilities. Traditionally LR has been widely used in clinical audit and the development and validation of mortality prediction models | [100,101] |
|  | Naïve Bayes classifiers | NBs is a classifier algorithm that assumes that a particular feature in a class is independent of any other feature | [102] |
|  | Random Forrest | A tool used for ensemble learning. RF uses a combination of DT predictors such that each tree depends on the values of a random vector sampled independently which in turn provides a majority “vote” to classify the outcome | [103] |
|  | Support Vector Machine | A powerful method that classifies data by defining a hyperplane that best differentiates two groups. The hyperplane-bounded region with the largest possible margin is used for analysis. SVM is particularly useful to find non-linear relationships through the use of a kernel function | [104] |
| Unsupervised learning | K-means clustering | An unsupervised method that uses discrete or continuous data as its input parameter for identifying input regularities (i.e. clusters) | [99] |
